# Dopamine-mediated photothermal theranostics combined with up-conversion platform under near infrared light

**DOI:** 10.1038/s41598-017-13284-5

**Published:** 2017-10-19

**Authors:** Ruichan Lv, Piaoping Yang, Guanying Chen, Shili Gai, Jiating Xu, Paras N. Prasad

**Affiliations:** 1 Key Laboratory of Superlight Materials and Surface Technology, Ministry of Education, College of Material Sciences and Chemical Engineering, Harbin Engineering University, Harbin, 150001 P. R. China; 2 Institute for Lasers, Photonics, and Biophotonics and Department of Chemistry, University at Buffalo, State University of New York, Buffalo, New York, 14260 United States; 3 School of Chemistry and Chemical Engineering, Harbin Institute of Technology, Harbin, 150001 P. R. China; 4 Engineering Research Center of Molecular and Neuro Imaging, Ministry of Education, School of Life Science and Technology, Xidian University, Xi’an, Shanxi 710071 China

## Abstract

An organic-inorganic hybrid core-shell nanostructure, based on mesoporous silica coated upconversion core-shell nanoparticles (NaGdF_4_:Yb,Er@NaGdF_4_:Yb@mSiO_2_-Dopa abbreviated here as UCNP@mSiO_2_-Dopa) that stably incorporates dopamine (Dopa) in the silica layer was introduced as a theranostic nanoplatform for optical imaging guided photothermal therapy (PTT) using NIR excitation. Silica-attaching polyethylenimine make the Dopa transforms into an active form (transferred Dopa) that strongly absorbs light under single 980 nm irradiation. We show that the activated UCNP@mSiO_2_-Dopa nanoplatform is able to produce a pronounced photothermal effect, that elevates water temperature from room temperature to 41.8 °C within 2 minutes, while concurrently emitting strong upconverted luminescence (UCL) for visualized guidance under 980 nm laser. In addition, we demonstrate the application of the same UCNP@mSiO_2_-Dopa nanoplatform for magnetic resonance imaging (MRI) and x-ray computed tomography (CT) enabled by the gadolinium (Gd) element contained in the UCNP. Importantly, the *in vitro* and *in vivo* anti-cancer therapeutic effects have been shown efficacious, implying the use of the described nanoplatform as an effective multi-modal imaging enabled PTT agent. Results from the *in vivo* biodistribution of UCNPs@mSiO_2_, cellular live/dead assay, and histologic analysis of main organs of treated mice, reveal that the UCNP@mSiO_2_-Dopa agents are bio-compatible with low toxicity.

## Introduction

Recently, light-activated cancer treatment has attracted considerable attention, as it can allow noninvasive regulation of the therapeutic process at the intended site, with a defined manner, thus overcoming the commonly met side effects (the undesired drug release, the drug resistance, *etc*) associated with conventional chemo-therapy^1–4^. Photothermal therapy (PTT) is such a technique that engages the use of agents to strongly absorb the electromagnetic radiation and then convert it nonradiatively to heat to kill targeted cancer cells^5–7^. Current PTT agents can be classified into four types: (i) noble metal nanoparticles, (ii) carbon complex, (iii) organic materials, and (iv) semiconductor naomaterials^8–12^. In particular, there have been major efforts in the development of PTT agent using near-infrared (NIR) light that is less absorbed in cells and tissues, thus providing deeper penetration to treat thick tumors, and minimizing collateral damage. Despite recent successes in development of PTT agents, it still remains a daunting challenge to find a single theranostic nanoplatform that can concurrently and effectively deliver both imaging and therapeutic abilities. Of the four types described above, PTT using metal nanoparticles, particularly gold nanostructures^13–17^, pioneered by Halas and co-workers^18^, El-Sayed and co-Workers have received most attentions^19^. They have proven to be highly effective nanoheaters using NIR excitation to allow treatment of thick tumor, and there are several ongoing clinical trials using nanogold for PTT, establishing its viability for cancer therapy in patients (Clinical Trial Phase I, NCT00356980, NCT00436410 from Cytimmune; Clinical Trial Phase I, NCT00848042, NCT01679470 AuroLase®). Although some modalities of imaging can utilize gold nanostructures itself, others may require additional surface modifications with dye and other optical agents for combined diagnostics and therapy. Thus, there is still a need to develop theranostic agents for PTT with optical and/or multi-modal imaging abilities which can allow high sensitivity, high accuracy, and depth-unlimited diagnosis, along with an effective PTT therapy^20–23^.

Toward this end, a straightforward way is to combine the optical imaging contrast agent and the PTT agent that can both be activated in the NIR range^24–26^. Upconversion nanoparticles (UCNPs) have shown to be an effective optical imaging contrast agents for use in biology due to their unique capability to convert NIR light into upconversion luminescence (UCL) at a number of wavelengths for multi-channel visualization^27–38^. The use of real ladder-like energy levels of lanthanide ions incorporated at the host lattice of UCNPs allows them to produce high efficiency UCL of light from a continuous-wave NIR diode laser or even an incoherent light source, facilitating their popular uses in biology^39,40^. Also, UCNPs show superior features such as high photochemical stability, low toxicity, non-blinking, non-photobleaching, absence of autofluorescence, deep tissue penetration, and inherent multimodal imaging abilities empowered by the composition^41–46^. Attempts have been made in the past years to combine the NIR-induced UCNPs and with other photo-active agents such as organic chemo-drugs through co-encapsulation of them into either an inorganic or organic shell layer^47–54^. However, two problems remain till this point: (i) the limited loading capacity of the photoactive agents and chemo-drugs; (ii) The premature leak-out and aggregation of the incorporated photoactive agents. It has been shown that the mesoporous silica is able to hold a substantial amount of therapeutic agents for controllable drug release, showing superior merits over the conventional organic drug delivery systems (DDSs)^55–58^. Yet, the combined use of mesoporous silica and UCNPs to enable UCL imaging-guided PTT has not been explored, mainly because of the lack of biocompatible organic PTT agents that are able to produce an effective interaction with UCNPs at NIR light excitation.

Dopamine (3,4-dihydroxyphenethylamine, Dopa) is an organic chemical of the catecholamine and phenethylamine families that plays important roles in the brain and body. It could function as a neurotransmitter or as a chemical messenger to detect the Parkinson’s disease. More importantly, owing to the aromatic ring and functional group of amino and hydroxyl, the dopa monomer as the organic precursor could be transferred to a melanin-like product that is widely contained in human organs and tissues, and that is able to strongly absorbs light. It has been shown that the melanin-like polydopamine nanoparticles could efficiently convert light into heat, suggesting its potential use as photothermal agent^59,60^. Moreover, melanin-like biopolymers have excellent biocompatible and biodegradable property as their component, Dopa, is a natural product of human beings^61–63^. However, till this point, there has been no report on combining UCNPs with dopamine and mesoporous silica to generate melanin-like PTT agent for image-guided theranostic applications in the NIR range.

**Figure 1:**
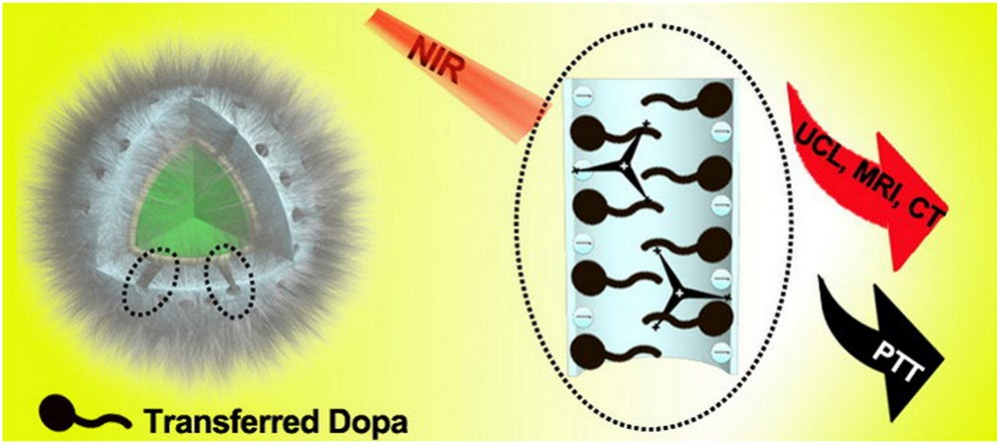
A schematic illustration of the acting principle of the organic-inorganic hybrid core/shell nanostructure of UCNP@mSiO_2_-Dopa.

Herein, we introduce an alternative PTT agent that utilizes an inorganic-organic hybrid nanostructure, involving DOPA in a core-shell geometry. Our design concept is illustrated in Fig. 1: 1) Dopa is transformed into a deprotonated form when incorporated into a mesoporous silica as the shell layer on the UCNP core, with the assistance of polyethylenimine (PEI). 2) The transferred Dopa could absorb the blue and green upconversion generated under 980 nm laser, and the unabsorbed red UCL from the UCNP provides optical imaging and tracking capability. Especially, this nanoplatform is able to produce a pronounced photothermal effect under 980 nm laser. 3) Owing to the high atomic number of Gd, the inorganic UCNP can also serve as a contrast agent for T1-weighted contrast of magnetic resonance imaging (MRI) and computed tomography (CT).

Specifically, a set of step-dependent experiments has been performed to reveal the evolution of the transferred-Dopa as PTT agent with the assistance of polyethylenimine (PEI). A comparison of the PTT effect of UCNPs@mSiO_2_-Dopa with UCNPs@mSiO_2_ demonstrates the important role of transferred-Dopa. Also, effective PTT of UCNP@mSiO_2_-Dopa was proved by both *in vitro* and *in vivo* experiments, along with their potential uses in UCL optical imaging, MRI, and CT. To assess the potential of the UCNP@mSiO_2_-Dopa for uses in clinical application, the bio-compatibility properties were evaluated by the *in vivo* biodistribution of UCNP@mSiO_2_ incorporating NIR indocynine (ICG) molecules for optical tracking, by live/dead assay, and by long-term (up to 21 days) histological analysis of main organs derived from the treated mice.

## Results and Discussion

### Formation, phase, structure, shape, and luminescent properties

**Figure 2:**
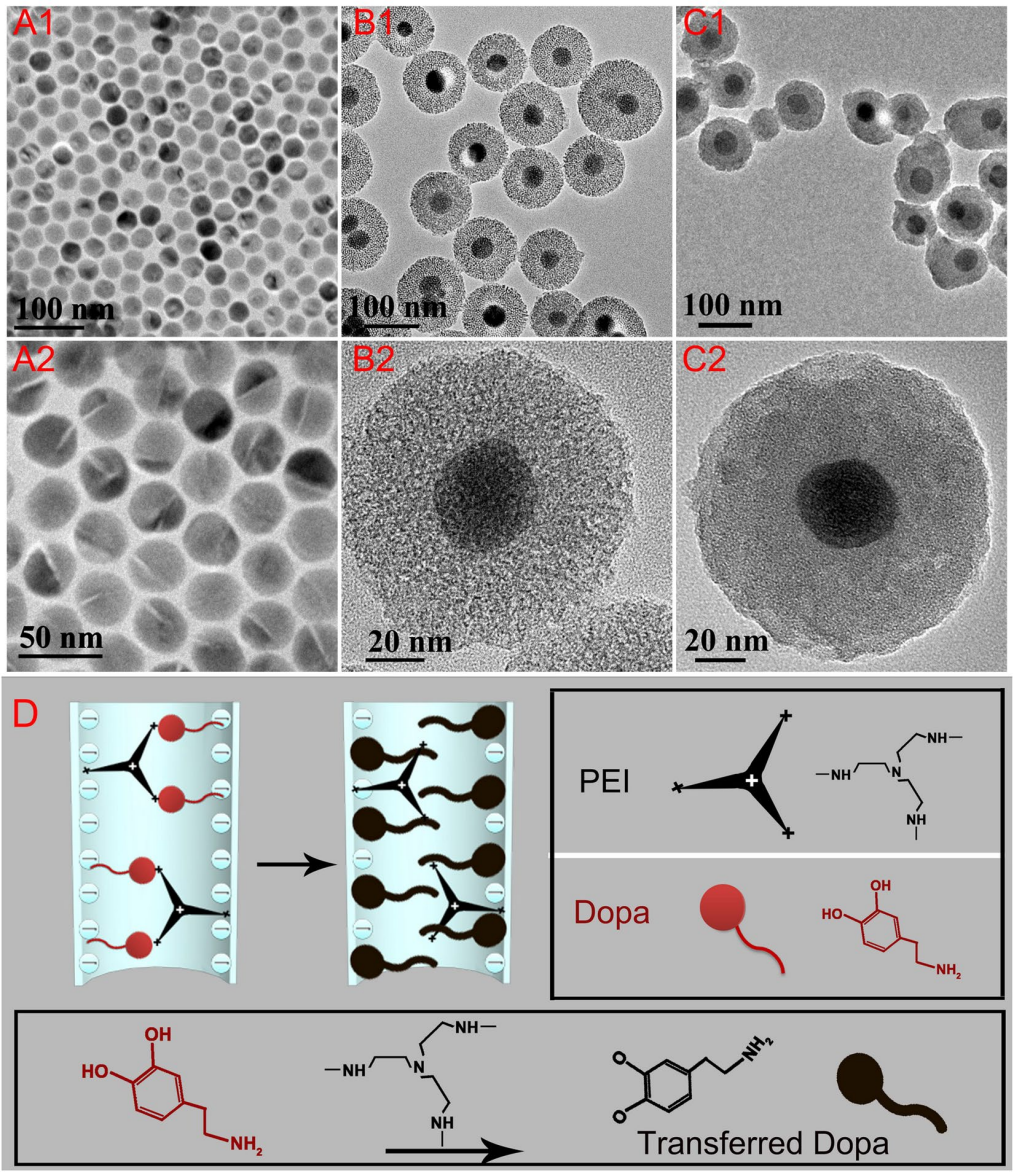
TEM images of (**A1**,**A2**) UCNPs, (**B1**,**B2**) UCNP@mSiO_2_, and (**C1**,**C2**) UCNP@mSiO_2_-Dopa. (**D**) A schematic diagram portraying the incorporation and transformation of the Dopa with the assistance of PEI polymer.

The XRD pattern of the as-synthesized UCNP@mSiO_2_-Dopa is shown in Figure S1, in which all diffraction peaks can be well assigned to hexagonal NaGdF_4_, revealing the hexagonal crystallographic phase of the core/shell NaGdF_4_:18%Yb,2%Er @NaGdF_4_:10%Yb UCNP. In addition, TEM images of the core/shell UCNPs are shown in Fig. 2A1 and A2. From these Figures, we can see that the as-synthesized core/shell UCNPs are uniform with an average size of 35 nm. TEM images of the UCNP@mSiO_2_ (further coated with a mesoporous silica shell layer) with different magnification times are shown in Fig. 2B1,B2 and Figure S2. As shown, the resulting UCNP@mSiO_2_ particles are monodisperse with an average total size of 110 nm. Porous cavities and channels in the silica shell can be clearly seen. TEM images of resulting UCNP@mSiO_2_-Dopa after further modification with PEI and Dopa are displayed in Fig. 2C1 and C2. As shown, the channels and cavities are sealed with transferred Dopa. A schematic carton is shown in Fig. 2D, illustrating the electrostatic stabilization of PEI (positive charge) into the mesoporous silica (negative charge), the incorporation of Dopa into the channel, and the transformation process of Dopa inside the mesoporous channel. As depicted, transferred Dopa could be generated under an alkaline environment provided by the amino-rich PEI polymer.

**Figure 3:**
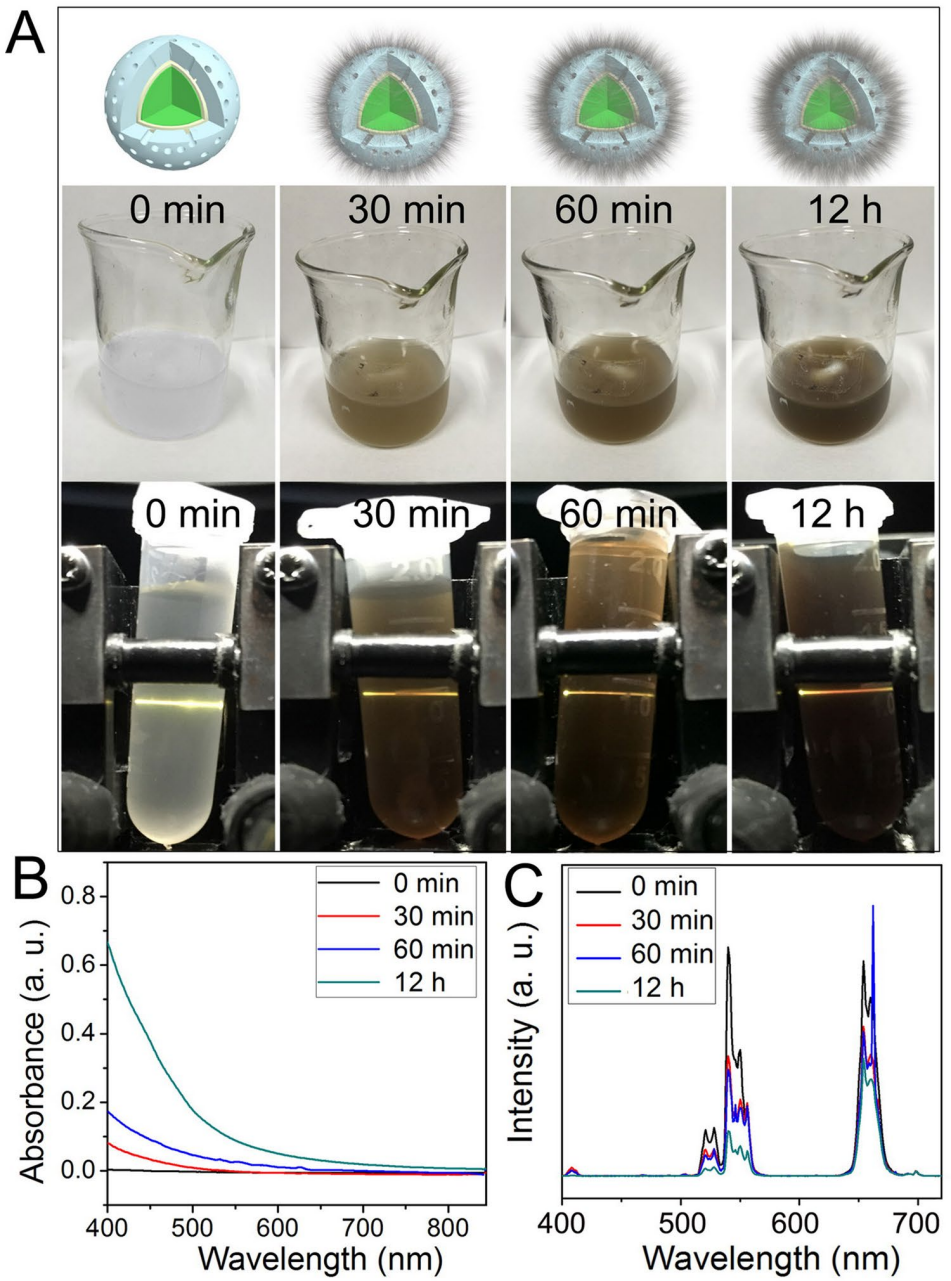
(**A**) Schematic diagrams, images of solution under daylight and under 980 nm irradiation with different reaction time: 0 min, 20 min, 60 min, and 4 h. (**B**) Absorbance spectra and (**C**) UCL spectra of the solutions with different reaction times under 980 nm irradiation.

To illustrate how the transferred Dopa could be generated as the PTT agent, a step-dependent experiment was carried out with defined reaction times of 0 min (UCNP@mSiO_2_), 30 min, 60 min, and 12 h (UCNP@mSiO_2_-Dopa). As shown in Fig. 3A, the color of the transparent solution gradually changed from colorless to dark, indicating that the –OH group in Dopa has been deprotonated to –O with prolonging reaction time. The colorless transparent solution at the initial stage indicates negligible absorbance from the mixture of pure UCNP@mSiO_2_ and pure Dopa, while the increased darkness of solution with reaction time implied the controllable growth of transferred Dopa under a modest alkaline environment inside the surface of the mesoporous silica. In fact, after centrifugation, the powders are changed from white to pale and pale-black, further indicating there is real transferred dopamine absorbed inside of mesoporous channels and surface. The UV-vis absorbance spectra of the corresponding solutions were collected and are presented in Fig. 3B. As shown, there are strong absorption peaks in the visible region (shorter than 600 nm) for samples with long reaction time.

Under 980 nm irradiation, the UCL color of the solution was converted from strong green to yellow/red with the prolonged reaction time, correlated with the darkening of the solution. We have also acquired the UCL spectra of the solution at different reaction time, and shown them in Figs 3C and S3A. As one can see, the UCL peaks at 409 nm (^2^H_9/2_ → ^4^I_15/2_) and 543 nm (^2^H_11/2_/^4^S_3/2_ → ^4^I_15/2_) from Er^3+^ decreased, while there is almost no reduction of UCL in the red region (^4^F_9/2_ → ^4^I_15/2_). The UCL intensity ratio of the UCNP@mSiO_2_-Dopa solution with different reaction time points is displayed in Figure S3B, which utilizes the luminescence intensity of the solution reacted at 12 h as a benchmark. The blue, green, and red UCL bands are decreased about ~12.7, 5.1, and 1.8, respectively. Meanwhile, the decay curves of UCNP@mSiO_2_-Dopa solution at emission wavelengths of 409, 543, and 650 nm with different reaction times are shown in Figure S4. The lifetimes at 409 nm decreased from 0.225 to 0.204 ms, while the lifetimes at 543 nm decreased from 0.366 to 0.313 ms, respectively. The shortening of the lifetime suggests that the energy transfer at the states of ^2^H_9/2_ and H_11/2_/^4^S_3/2_ from UCNPs to the transferred Dopa is a non-radiative luminescence resonance energy transfer process (LRET). It should be noted that there is almost no decrease in the lifetime at 650 nm within 1 h (0.400 ms to 0.403 ms), indicating the energy transfer from the ^4^F_9/2_ level to Dopa is rather limited. Taken together, besides of the self-absorption of UCNP@mSiO_2_-Dopa under 980 nm irradiation, the upconverted energy from the ^2^H_9/2_ and ^2^H_11/2_/^4^S_3/2_ excited levels of Er^3+^ can be also transferred to Dopa with strong absorbance at the wavelength shorter than 600 nm, producing a PTT effect simultaneously.

**Figure 4:**
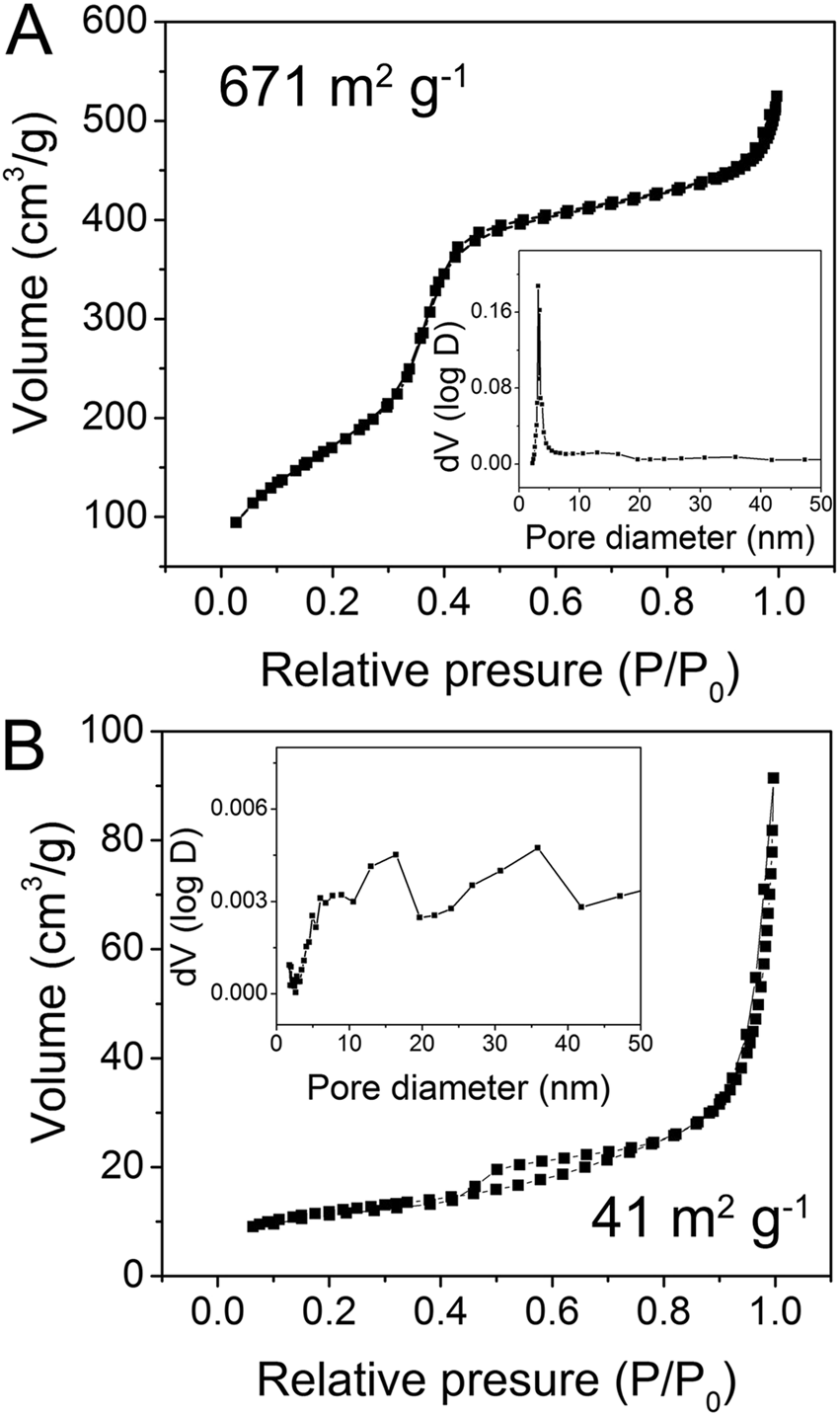
N_2_ adsorption/desorption curves of (**D**) UCNP@mSiO_2_ and (**E**) UCNP@mSiO_2_-Dopa with pore volume distributions inset.

**Figure 5:**
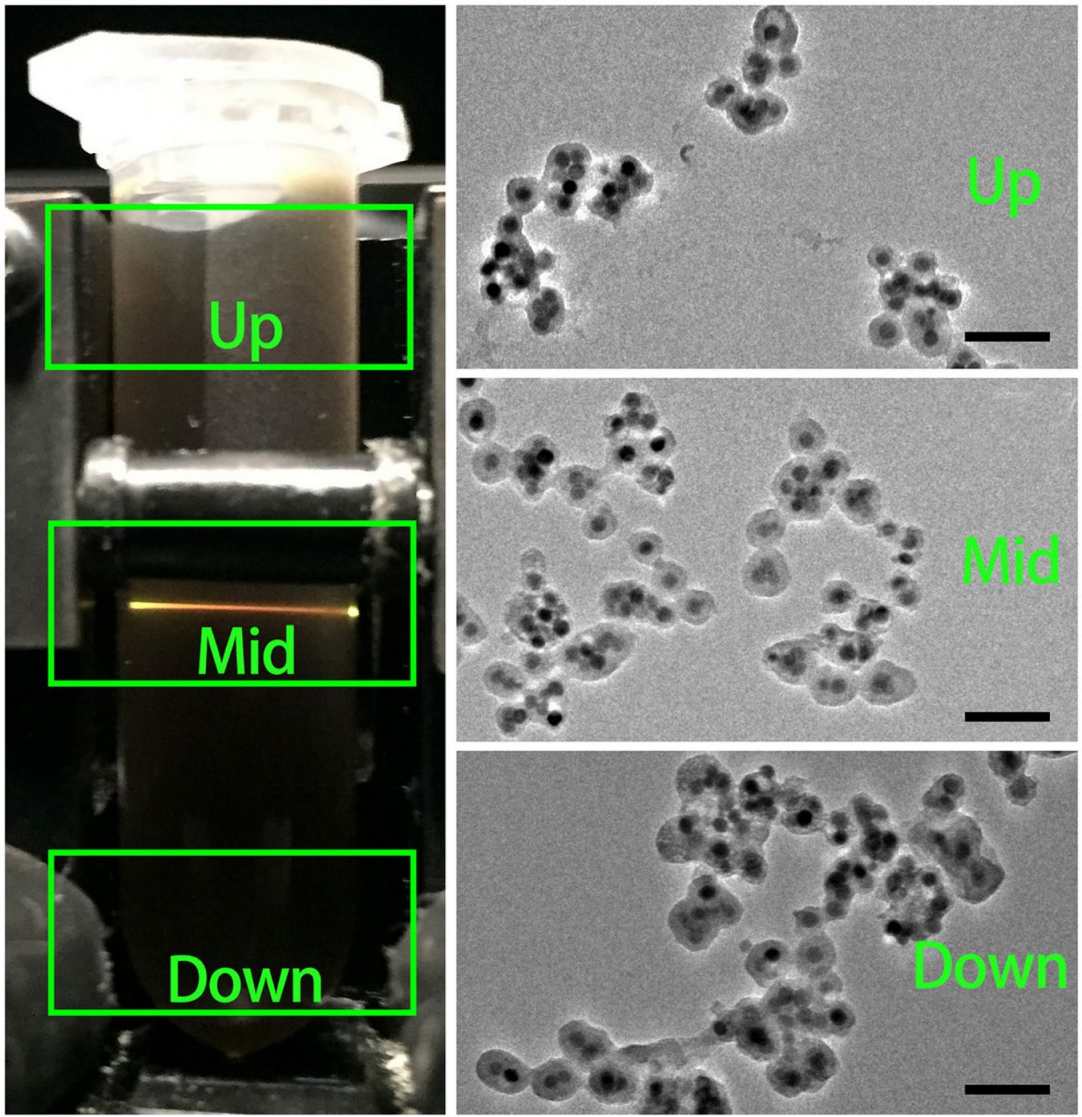
The stability properties of UCNP@mSiO_2_-Dopa solution. Photograph and TEM images of the solution after 2 days. All of the scale bars of TEM images are 200 nm.

N_2_ adsorption/desorption curves of UCNP@mSiO_2_ and UCNP@mSiO_2_-Dopa are presented in Fig. 4A and B, revealing a reduction of the pore size due to the incorporation of Dopa. The BET surface area and the pore volume of UCNP@mSiO_2_ are 671 m^2^ g^−1^ and 1.027 cm^3^ g^−1^, respectively. As shown in the pore volume distributions inset, there are regular mesoporous pores and channels distributed in the silica structure. After the formation of transferred Dopa, the BET surface area and pore volume of UCNP@mSiO_2_-Dopa are decreased to 41 m^2^ g^−1^ and 0.12 cm^3^ g^−1^, respectively. Meanwhile, there is no main peak with the pore diameter under 50 nm. The sharply decreased pore volume is attributed to the occupation of the transferred Dopa in mesoporous silica channels, agreeing well to the TEM observations in Fig. 2C1 and C2. In addition, TEM images of different parts of UCNP@mSiO_2_-Dopa solution (being placed for 2 days) are measured (Fig. 5), which shows no further aggregation in the solution, implying that the generated PTT agents are uniform and have good colloidal stability.

### Photothermal anti-cancer theranostic effect

**Figure 6:**
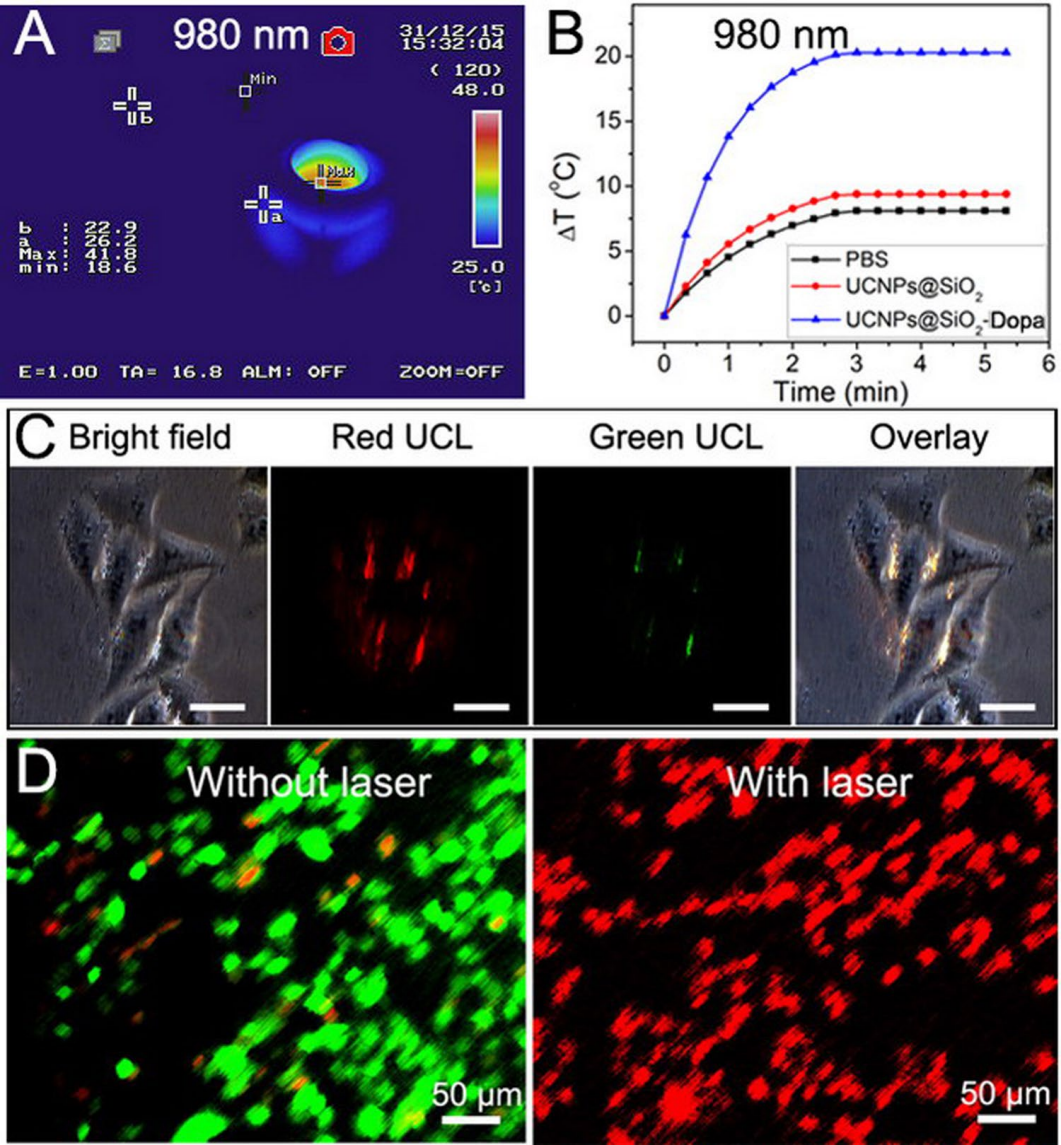
(**A**) The infrared thermal image of UCNP@mSiO_2_-Dopa solutions under 980 nm laser. (**B**) The temperature curves of UCNP@mSiO_2_-Dopa solution under 980 nm laser. (**C**) The inverted luminescence microscopy image of HeLa cells incubated with UCNP@mSiO_2_-Dopa for 3 h. (**D**) The live/dead state of HeLa cells incubated with UCNP@mSiO_2_-Dopa with and without 980 nm irradiation.

To evaluate the real temperature change in the aqueous solution induced by the as-synthesized PTT agent, we recorded the thermal imaging photos under excitation with different solutions: PBS saline solution, PBS solution containing UCNP@mSiO_2_ and UCNP@mSiO_2_-Dopa (Figure S5). Note that the UCNP@mSiO_2_ and UCNP@mSiO_2_-Dopa have the same amount with the concentration of 1 mg/mL. As shown, temperature of UCNP@mSiO_2_-Dopa increased from room temperature to 41.8 °C under the 980 nm irradiation (Fig. 6A). As a control, there is also no apparent temperature increase in the other two solutions of PBS saline solution, PBS solution containing UCNP@mSiO_2_ (Fig. 6B). This indicates that the UCNP@mSiO_2_ without the transferred Dopa could not be used as a PTT agent.

**Figure 7:**
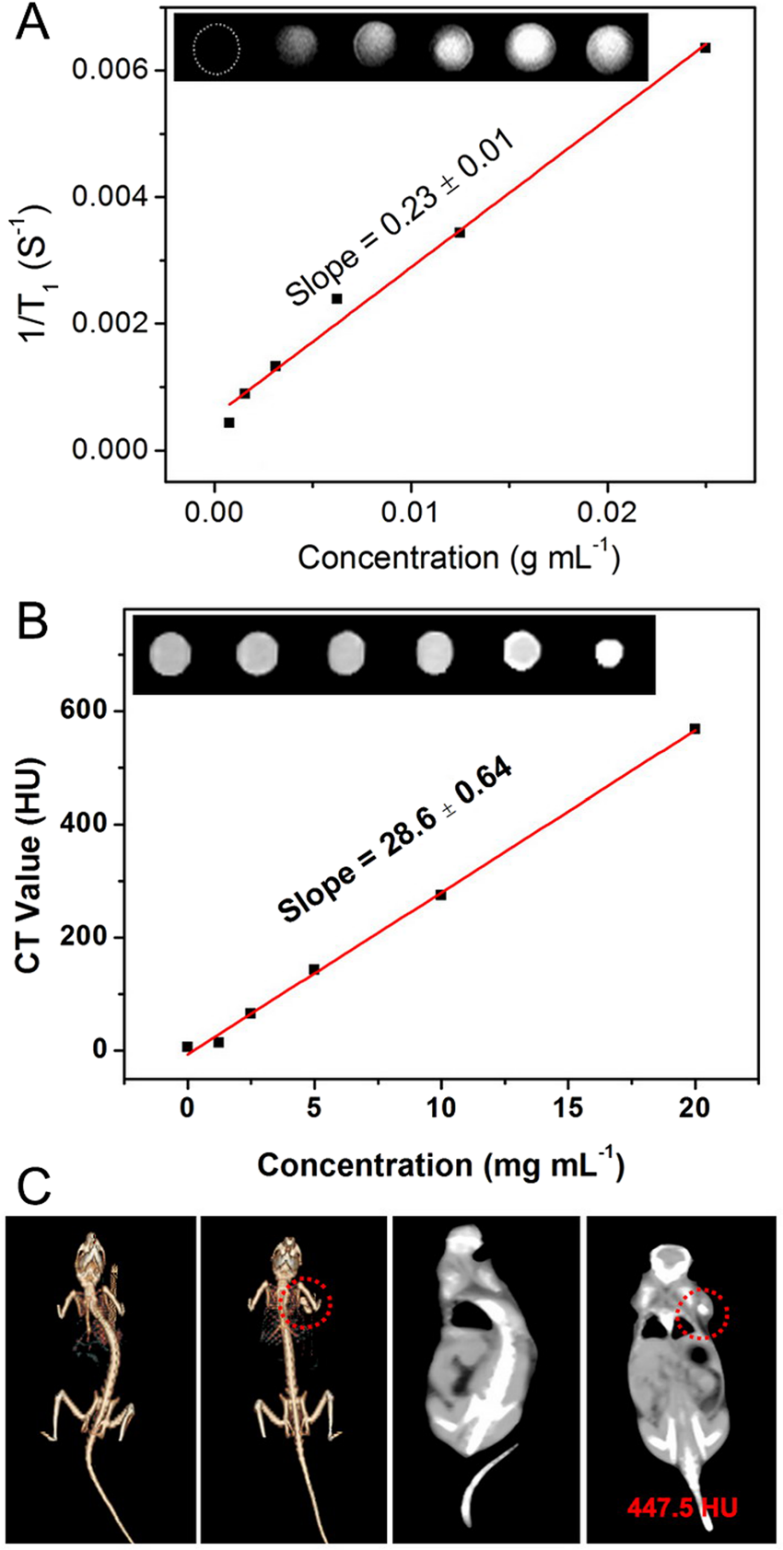
The *in vitro* (**A**) MRI and (**B**) CT images corresponding value as a function of the concentration of UCNP@mSiO_2_-Dopa. (**C**) *In vivo* CT images of tumor-bearing Balb/c mouse before and after injection of UCNP@mSiO_2_-Dopa.

When HeLa cells were incubated with UCNP@mSiO_2_-Dopa for 3 h, the intracellular uptake of the material could be clearly seen (Figure S6). Meanwhile, under 980 nm irradiation, the red/yellow luminescence could be detected (Fig. 6C). Along with optical imaging, the MRI and X-ray CT have been proved to be effective diagnostic imaging techniques due to their high resolution imaging ability in deep tissue, providing complimentary anatomic information to optical imaging. Gd-containing particles can be potentially used as contrast agents for both MRI and CT imaging. Indeed, as shown in Fig. 7A, the longitudinal (*r*_1_) relaxivity value of our UCNP@mSiO_2_-Dopa sample was measured to be 0.23 mL g^−1^ S^−1^, implying its use as MRI agent. While as shown in Fig. 7B, the CT signal intensity clearly increases with increased concentration of UCNP@mSiO_2_-Dopa, exhibiting a large slope of 28.6. The *in vivo* CT imaging ability was further evaluated by intratumoral injection of our UCNP@mSiO_2_-Dopa sample (Fig. 7C); the CT value in the tumor area can reach up to 447.5 HU (Hounsfield units). Taken together, the imaging results clearly show that UCNP@mSiO_2_-Dopa samples can be potentially utilized as an effective contrast agent for UCL, MRI, and CT tri-modal imaging.

The live/dead state of HeLa cells incubated with UCNP@mSiO_2_-Dopa, with and without 980 nm irradiation, is presented in Fig. 6D. The cells are marked with calcein AM (dyed the live cells into green) and PI (dyed the dead cells into red). It can be seen that almost all the cells are alive before laser treatment, indicating the UCNP@mSiO_2_-Dopa agent are bio-compatible with low toxicity. When the nanoparticle-treated HeLa cells were exposed to 980 nm irradiation for 10 min, nearly all the cancer cells have been killed (right image in, implying the use of UCNP@mSiO_2_-Dopa as the photothermal agent for anti-cancer therapy, while with red UCL for guidance under one single NIR wavelength excitation.

**Figure 8:**
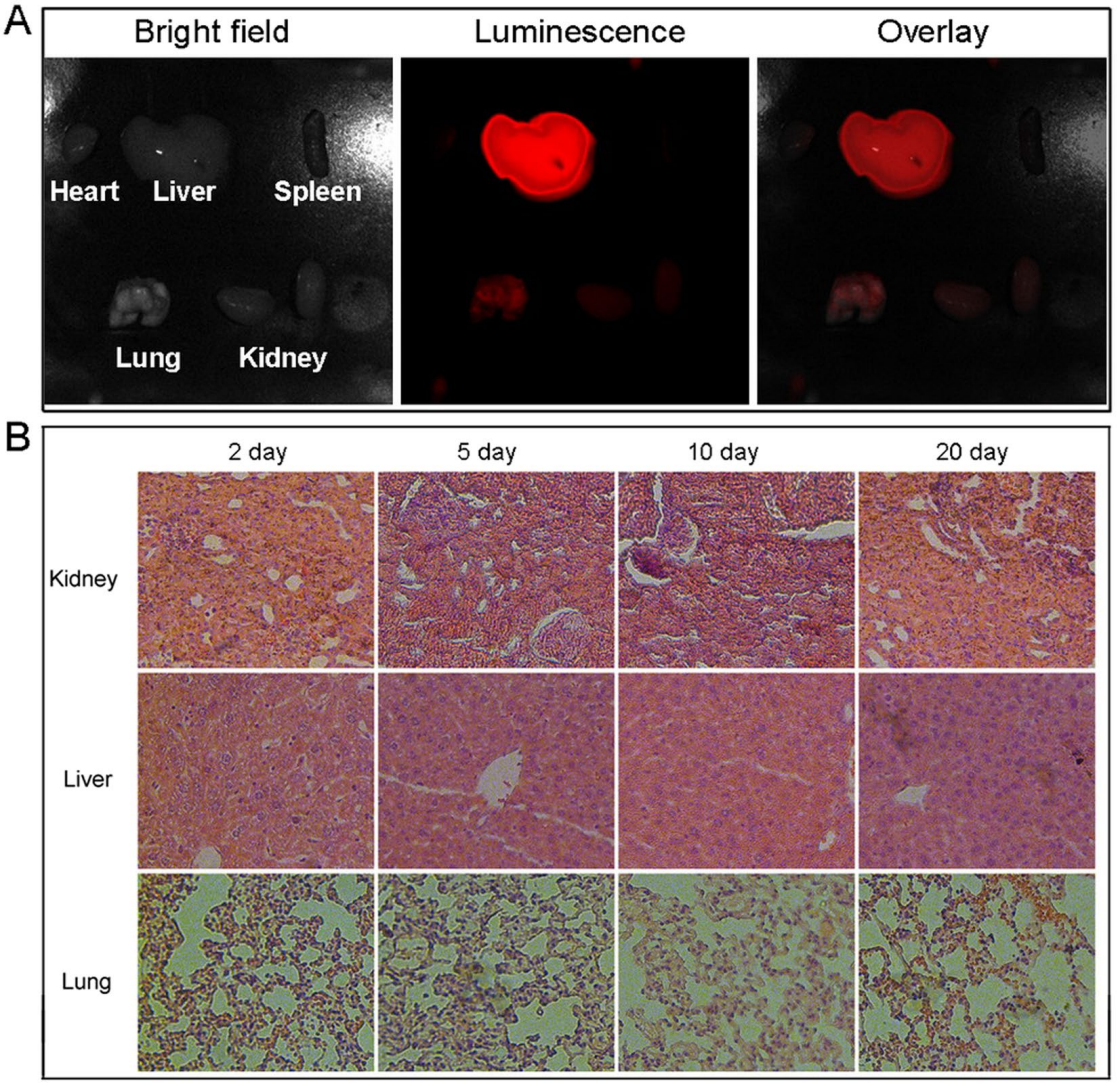
(**A**) The luminescence imaging showing nanoparticle bio-distribution in main mouse organs after intravenous injection of UCNP@mSiO_2_ incorporating ICG molecules for 2 h. (**B**) H&E stained images of the kidney, liver, and lung after injection of UCNP@mSiO_2_-Dopa for different days.

To show the potential of the UCNP@mSiO_2_-Dopa for uses in clinical application, the bio-compatibility and bio-degradability properties are evaluated. Figure 8A show the bio-distribution of the UCNP@mSiO_2_ sample in the major organs of a mouse after intravenous injection for 2 h. Here, the NIR indocynine (ICG) molecules have been incorporated into the mesoporous silica to enable optical tracking (excited at 740 nm, detection at wavelengths over 800 nm) of these nanoparticles using the commercial Magestro imaging setup. There are three reasons that we chose UCNP@mSiO_2_ instead of UCNP@mSiO_2_-Dopa to show the bio-distribution properties: (a) The ICG dye was proposed as an ideal optical tracking agent to show the bio-distribution of the nanoparticles. UCNP@mSiO_2_ is difficult to see the organs of mouse which have much lower penetration depth than ICG dye, so we would use ICG as the imaging agent to the deep organs of mice. (b) When the ICG molecules were needed to be incorporated into the nanoparticles, the carrier should be with high mesoporous surface area. N_2_ adsorption/desorption curves of UCNP@mSiO_2_ and UCNP@mSiO_2_-Dopa suggest that the surface area of UCNP@mSiO_2_ is much higher, so the carrier should be UCNP@mSiO_2_. (c) It is well known that the melanin-like dopamine and transferred dopamine are excellently biocompatible as the natural part of human being, so there was almost no difference to use the UCNP@mSiO_2_ in place of UCNP@mSiO_2_-Dopa. As shown in Figure S7, these particles are distributed in the liver, lung, and kidney. Pathologic analysis results are shown in Fig. 8B; there is no obvious abnormal in the three main organs of kidney, liver, and lung after 21 days, suggesting the low toxicity of UCNP@mSiO_2_-Dopa and the nanoparticles could be biodegradable by liver or kidney. PTT anti-tumor performance of these nanoparticles in mouse was further examined.

**Figure 9:**
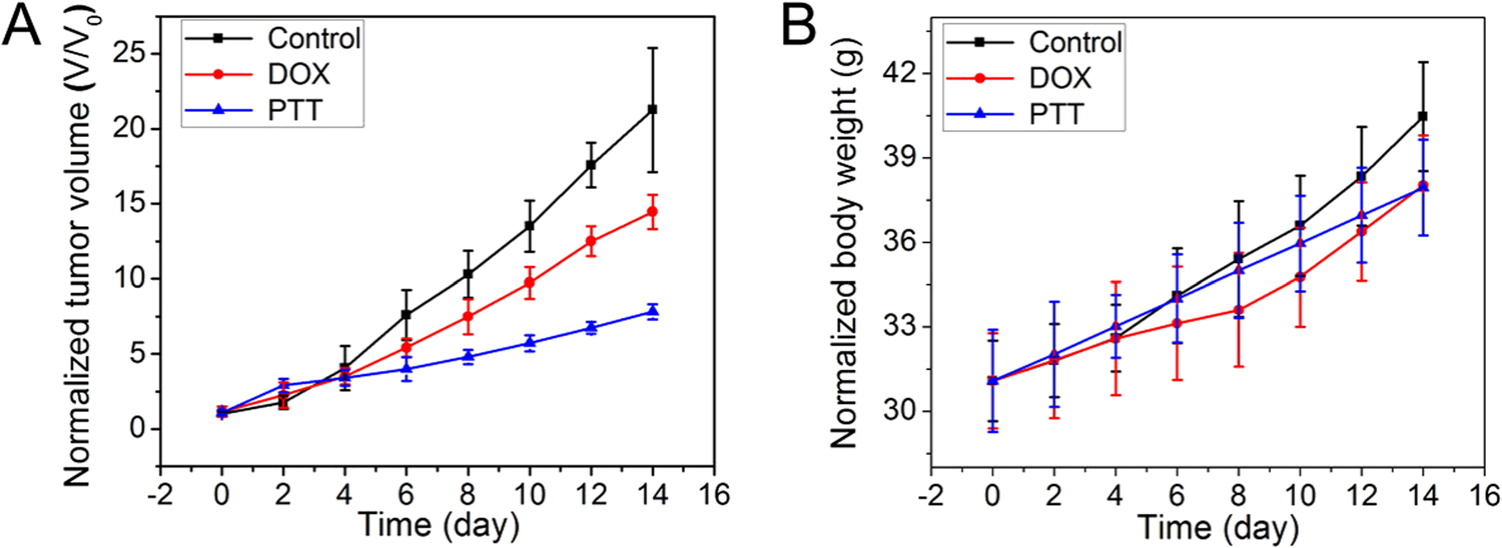
(**A**) The tumor volume and (**B**) body weight of mice treated with different groups: control, chemo-therapy treatment with pure DOX, and PTT treatment with UCNP@mSiO_2_-Dopa under NIR laser.

The tumor-bearing mice were divided into three groups: the blank group without any treatment as control, the group treated with pure doxorubicin (DOX) as chemo-therapy control group, and the group treated with UCNP@mSiO_2_-Dopa under NIR laser as PTT group. After treatment with designated conditions every two days for 14 days, the tumors from different mouse groups (under different treatment) were harvested and shown in the inset of Fig. 9A. As can be seen, the group with PTT treatment has the highest anti-cancer efficiency (the smallest tumor size), even more effective than the mouse group with chemotherapeutic treatment of DOX, a well-established chemotherapy agent. Meanwhile, the body weights of the mouse with PTT treatment keep increasing, indicating there is no side effect of UCNPs-Dopa agent (Fig. 9B). H&E stained images of the tissues from the three groups are presented in Figure S8. It is shown that there are no abnormal phenomena in the group treated with PTT: no damage is observed in the hepatocytes, no fibrosis appears in the pulmonary, and no concentration is found in the glomerulus. Both the *in vitro* and *in vivo* results evidently demonstrate the potential use of UCNP@mSiO_2_-Dopa as an effective PTT agent.

## Conclusions

In summary, we have designed a theranostic core/shell nanoplatform (UCNP@mSiO_2_-Dopa) for uses in red visible UCL guided PTT anti-cancer therapy through upconversion of a single NIR wavelength excitation at 980 nm. The transferred Dopa could be easily grown and sealed in the channel of the mesoporous silica layer without premature leakout, and the generated nanocomplex is stable and hydrophilic. Moreover, the tri-modal imaging ability of UCNP@mSiO_2_-Dopa for UCL, MRI, and CT have been demonstrated. Most importantly, under NIR irradiation at ~980 nm, UCNP@mSiO_2_-Dopa nanoplatform could produce a pronounced PTT effect that elevates water temperature from room temperature to 41.8 °C within just 2 min. Both the *in vitro* and *in vivo* results have shown the effectiveness of the UCNP@mSiO_2_-Dopa as a theranostic PTT agent, thus promises for their potential uses in personalized clinical applications.

## Experimental

### Reagents and materials

All chemical reagents used are analytical grade without any further purification, including polyethylene glycol (PEG500) which was purchased from Beijing Chemical Corporation, Beijing, China. Lanthanide oxides of Gd_2_O_3_ (99.99%), Yb_2_O_3_ (99.99%), Er_2_O_3_ (99.99%)_,_ and Nd_2_O_3_, (99.99%) were obtained from Sinopharm Chemical Reagent Co., Ltd., Shanghai, China. Cyclohexane, n-hexane, hydrochloric acid (HCl), cetyltrimethyl ammonium bromide (CTAB), sodium hydroxide (NaOH), tetraethoxysilane (TEOS), ammonium nitrate (NH_4_NO_3_), 1,4-dioxane, phosphate buffered saline (PBS), and the glutaraldehyde were obtained from Tianjin Kermel Chemical Reagent Co., Ltd., Tianjin, China. Oleic acid (OA, technical grade), 1-octadecene (ODE, technical grade), Polyethylenimine (PEI, mw = 1800), indocyanine green (ICG), Glycerol, calcein AM, and propidium iodide (PI) were ordered from Sigma-Aldrich, United States.

### Synthesis of OA-coated NaGdF_4_:Yb,Er

Firstly, the RE(OA)_3_ was prepared with the corresponding RECl_3_. In a typical process, 10 mmol of RECl_3_ was mixed with 30 mmol of sodium oleate, with 15 mL of deionized water, 20 mL of ethanol, and 35 mL of n-hexane. After backflow at 70 °C for 4 h, the mixture was further drying for 1.5 h to remove the survived liquid in a plate under water bath with temperature of 80 °C. Finally, RE(OA)_3_ was obtained through air drying under room temperature. 1 mmol of RE(OA)_3_ (RE = 18% Yb, 2% Er), 12 mmol of NaF, 15 mL of oleic acid (OA), and 15 mL of 1-octadecene (ODE) were all added into a three-neck reaction vessel, which was then heated to 110 °C under vacuum for 30 min to remove residual oxygen and water molecules. Subsequently, the temperature of the reaction system was raised to 310 °C at a rate of ~12 °C/min, and kept at this temperature for 1.5 h in N_2_ atmosphere. After naturally cooling down to room temperature, an excessive amount of ethanol (~30 mL) were added into the three-necked flask to precipitate the product of NaGdF_4_:Yb,Er nanoparticles. These nanoparticles were collected with centrifugation, and washed with ethanol and cyclohexane several times for purification. Finally, the NaGdF_4_:Yb,Er nanoparticles were dispersed in 10 mL cyclohexane for further use.

### Synthesis of OA-coated NaGdF_4_:Yb,Er@NaGdF_4_:Yb core/Shell nanoparticles

Firstly, the NaGdF_4_:Yb,Er core nanoparticles dispersed in cyclohexane was loaded to a three-neck reaction vessel, together with OA (15 mL), ODE (15 mL), Gd(CF_3_COO)_3_ (0.45 mmol), Yb(CF_3_COO)_3_ (0.05 mmol), and CF_3_COONa (1 mmol). The mixture was then heated to 120 °C under vacuum for 30 min to remove residual oxygen and water molecules. After that, the reaction temperature was elevated to 320 °C and maintained at this temperature for 1 h under N_2_ gas protection. After cooling down to room temperature, the resulting NaGdF_4_:Yb/Er@NaGdF_4_:Yb particles were collected through centrifugation, washed with ethanol and cyclohexane several times, and finally dispersed in cyclohexane (10 mL) for further use.

### Synthesis of hydrophilic UCNP@mSiO_2_

The as-prepared OA-coated nanoparticles (dispersed in 2 mL of cyclohexane from the original obtained 10 mL of cyclohexane in the last step) was first mixed with 20 mL deionized water and 0.2 g of CTAB under magnetic stirring for 24 h. During this process, cyclohexane was gradually removed, while the NaGdF_4_:Yb,Er@NaGdF_4_:Yb nanoparticles was stabilized by CTAB to enable them dispersed in water. Subsequently, 1 mL of ammonia was added into the solution to produce an alkaline environment. After that, the solution was placed in water bath at 70 °C. Then, 0.2 mL of TEOS was added, allowing its hydrolysis for 10 min. The mixture was then centrifuged and washed with water three times to get silica/CTAB coated UCNPs. To obtain a mesoporous silica coating layer, the solution was mixed with 50 mL of ethanol and 0.3 g of NH_4_NO_3_ under water reflux at 60 °C for 2 h to remove the CTAB in the silica shell. Finally, the mixture was washed with ethanol three times, and dispersed in water.

### Synthesis of UCNP@mSiO_2_-Dopa

The as-synthesized hydrophilic UCNP@mSiO_2_ was firstly stirred with 30 mL of PEI (concentration: 50 g L^−1^) for 4 h under room temperature. After purification through repeated centrifugation in deionized water, the powders were dissolved into deionized water. Then, 0.1 g of Dopa was added into the transparent solution, and the solution was stirred for 12 h which then turned dark. Finally, the product, denoted as UCNP@mSiO_2_-Dopa, was obtained through centrifugation with water for three times. Before bio-application, 20 mg of PEG was modified to the composite.

### Characterization

The X-ray diffraction (XRD) pattern was measured using a Rigaku D/max TTR-III diffractometer with graphite monochromatized Cu Kα radiation (λ = 0.15405 nm), and the scanning rate is 15° min^−1^ with 2θ range between 10° and 80°. The morphologies of the samples were examined by transmission electron microscopy (TEM, FEI Tecnai G^2^ S-Twin). Nitrogen adsorption/desorption isotherms were acquired on a Micromeritics ASAP Tristar II 3020 apparatus, and the pore size distribution was calculated by the Brunauer-Emmett-Teller (BET) method. UCL spectra were obtained using a 980 nm laser diode Module (MDL-III-980-2W) and recorded on a spectrofluorometer (Edinburgh FLS 980). The ultraviolet visible (UV-vis) absorbance spectra of the solutions were measured by a UV-1601 spectrophotometer. All the tests were carried out at room temperature, and all the methods were performed in accordance with the relevant guidelines and regulations of Harbin Engineering University and SUNY at Buffalo.

### *In Vitro* cellular uptake

The cellular uptake process was studied using HeLa cells. Briefly, the cells were cultured to get a monolayer in the 6-well plates with coverslips. Then, the cells were incubated with UCNP@mSiO_2_-Dopa at 37 °C for 3 h. After that, the well was washed with 1 mL of 2.5% glutaradehyde for fixation for 10 min. After further wash with PBS, the coverslip was recoded on Nikon Ti-S with an external 980 nm laser irradiation.

### Magnetic resonance imaging (MRI)

The test was carried out on a 0.5 T MRI magnet. UCNP@mSiO_2_-Dopa were dispersed in water with various concentrations of 25, 12.5, 6.25, 3.13, 1.56, and 0.78 mg mL^−1^. *T*he *r*_1_ relaxivity values were determined through a linear curve fitting of 1/*T*_1_ relaxation time (s^−1^) versus Gd concentration (g mL^−1^).

### X-ray computed tomography (CT) imaging

The *in vitro* CT imaging experiment was performed on a Philips 64-slice CT scanner at voltage of 120 kV. The UCNP@mSiO_2_-Dopa material was first dispersed in PBS with various concentrations of 20, 10, 5, 2.5, 1.25, and 0.63 mg mL^−1^, and then placed in a series of 2 mL tubes for CT imaging. For *in vivo* CT imaging, tumor-bearing Balb/c mice were first anesthetized with 10% chloral hydrate through an intra-peritoneal injection. Subsequently, 100 μL of UCNP@mSiO_2_-Dopa (20 mg/mL) was intratumorally injected into the mice *in situ*. The mice were scanned before and after injection of the sample.

### *In vitro* live/dead state detection

Two groups of HeLa cells were incubated with UCNPs (1 mg mL^−1^) for 3 h to allow efficient cellular uptake first. Then, the two cellular groups were treated without and with 980 nm irradiation (0.72 W cm^−2^), respectively. After irradiation for 10 min, the cells were dyed with calcein AM and PI at 37 °C for 1 h. Finally, the coverslips were washed with PBS, and cells were imaged by a confocal microscope (Leica TCS SP2).

### *In vivo* anti-cancer therapy

All the mouse experiments were performed in compliance with the criterions of The National Regulation of China for Care and Use of Laboratory Animals. Tumors were generated in the left axilla of each mouse (female Balb/c with weight of ~20–25 g) by subcutaneous injection of U14 cells. After growth for next 6 days, the tumor size is ~5–8 mm. The tumor-bearing mice were randomly divided into three groups (n = 6 per group): the control group, the chemo-therapy group with pure DOX, and the group with UCNP@mSiO_2_-Dopa under 980 nm irradiation (0.72 W cm^−2^), respectively. The mouse was injected every two days with 100 μL of DOX (0.1 mg mL^−1^) and UCNP@mSiO_2_-Dopa (1 mg mL^−1^). The tumor site was irradiated every two days for 14 days. The irradiations were carried out on the mice for 10 min each time.

### Histology examination

After treating for pre-set days (3, 7, 14, and 21 days), the representative organs of heart, liver, spleen, lung, and kidney tissues were harvested for histological analysis. The organs and tissues were dehydrated first. After that, the dehydrated tissues were embedded in liquid paraffin, and then sliced to 3–5 mm for hematoxylin and eosin (H&E) staining. Finally, the stained slices were placed on the coverslips and examined with a microscope.

## Electronic supplementary material

supporting information
