# Supplementary material for: Dopamine-mediated photothermal theranostics combined with up-conversion platform under near infrared light
Source: Sci Rep. 2017 Oct 19;7:13562. doi: 10.1038/s41598-017-13284-5 (PMC5648774; doi:10.1038/s41598-017-13284-5)
Supplement: Supplementary file 1 — supporting information [file 41598_2017_13284_MOESM1_ESM.doc]

**Dopamine-mediated photothermal theranostics combined with up-conversion platform under near infrared light**

**Ruichan Lv^1,2,4^, Piaoping Yang^1,*^,Guanying Chen^2, 3,*^, Shili Gai^1^, Jiating Xu^1^, Paras N. Prasad^2,*^**

^1^ Key Laboratory of Superlight Materials and Surface Technology, Ministry of Education, College of Material Sciences and Chemical Engineering, Harbin Engineering University, Harbin, 150001, P. R. China,

^2^ Institute for Lasers, Photonics, and Biophotonics and Department of Chemistry, University at Buffalo, State University of New York, Buffalo, New York 14260, United States

^3^ School of Chemistry and Chemical Engineering, Harbin Institute of Technology, Harbin, 150001, P. R. China

^4^ Engineering Research Center of Molecular and Neuro Imaging, Ministry of Education, School of Life Science and Technology, Xidian University, Xi’an, Shanxi 710071, China

* [yangpiaoping@hrbeu.edu.cn](mailto:yangpiaoping@hrbeu.edu.cn); [chenguanying@hit.edu.cn](mailto:chenguanying@hit.edu.cn); [pnprasad@buffalo.edu](mailto:pnprasad@buffalo.edu)


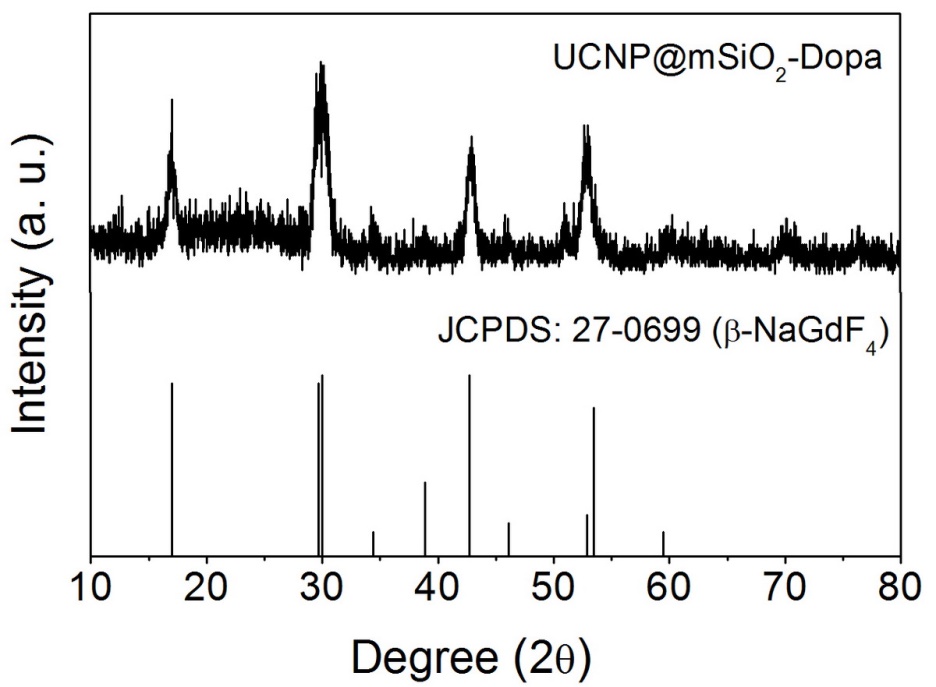


**Figure S1** XRD pattern of the as-synthesized UCNP@mSiO_2_-Dopa.


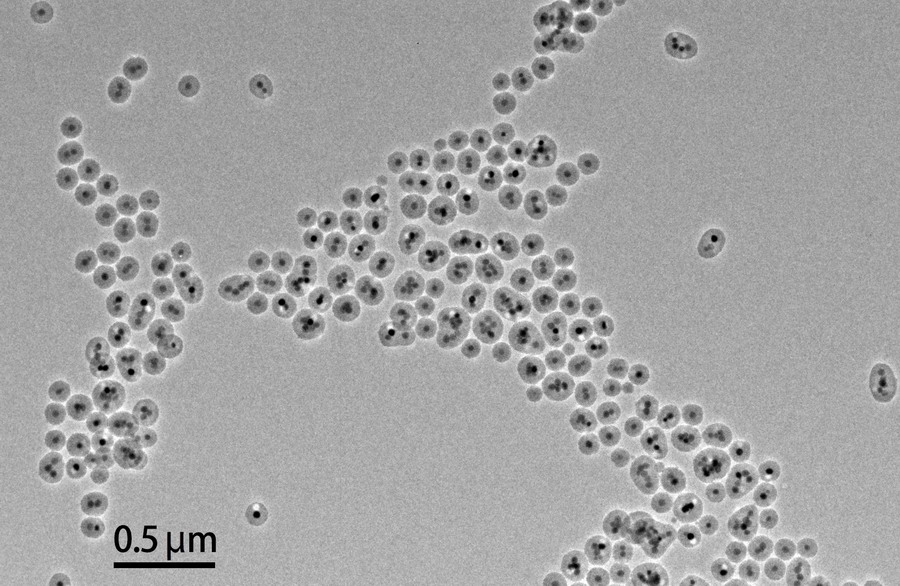


**Figure S2** TEM image of UCNP@mSiO_2_.


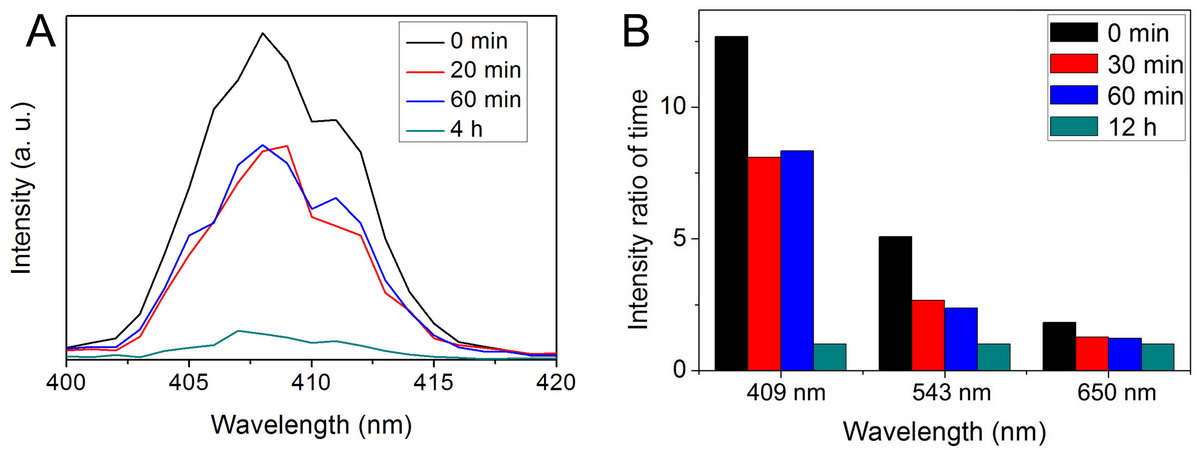


**Figure S3** (A) UCL emission spectra from 400 nm to 420 nm and (B) UCL intensity ratio of UCNP@mSiO_2_-Dopa solution with different reaction time points. (The intensity of solution reacted at 12 h is normalized.)


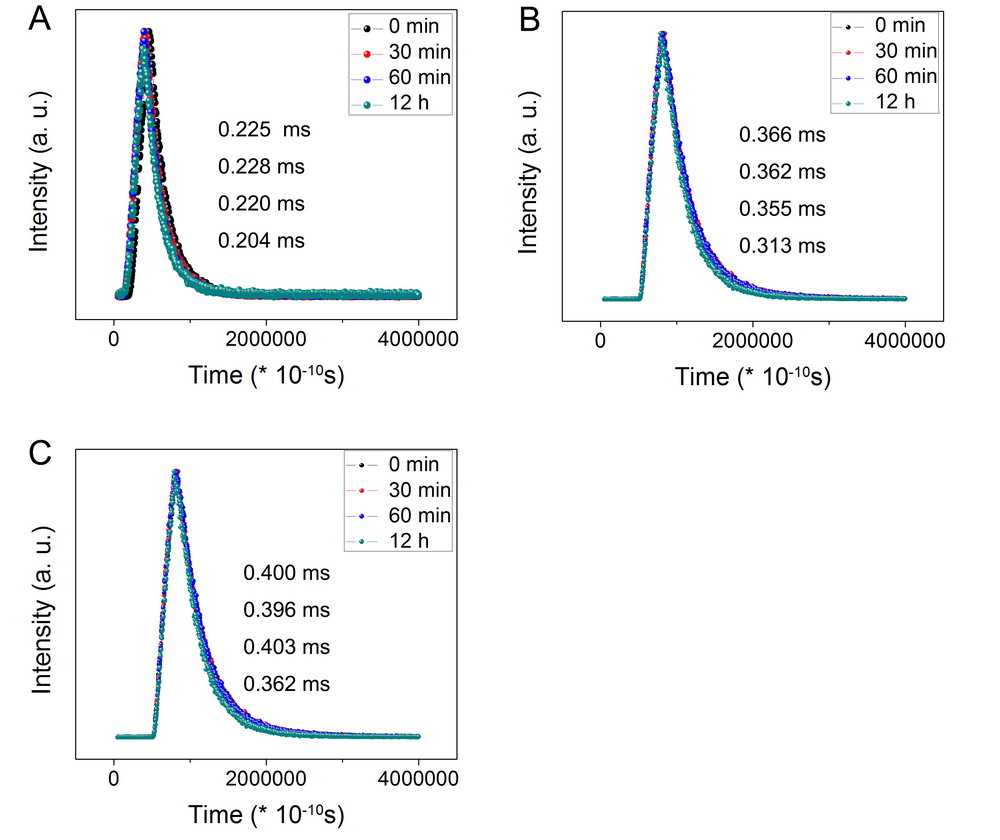


**Figure S4** The lifetimes of UCNP@mSiO_2_-Dopa solution with different reaction times.


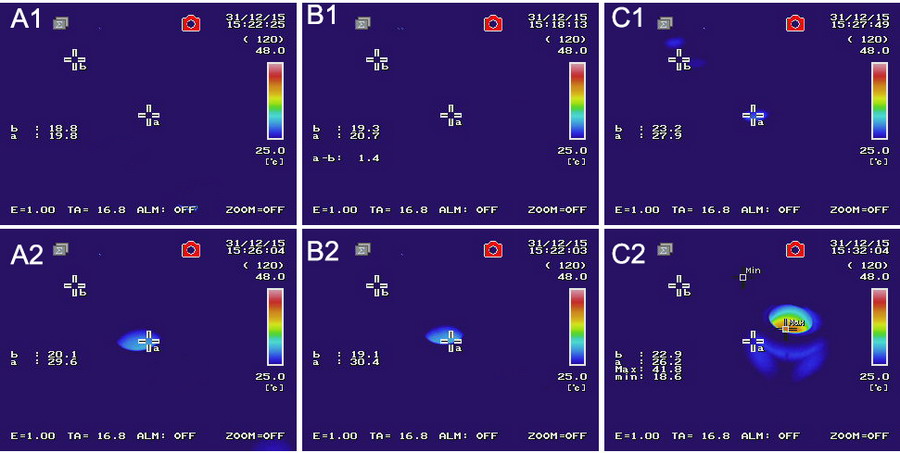


**Figure S5** The infrared thermal images of (A1, A2) PBS solution, (B1, B2) UCNP@mSiO_2_ solution, and (C1, C2) UCNP@mSiO_2_-Dopa solution.


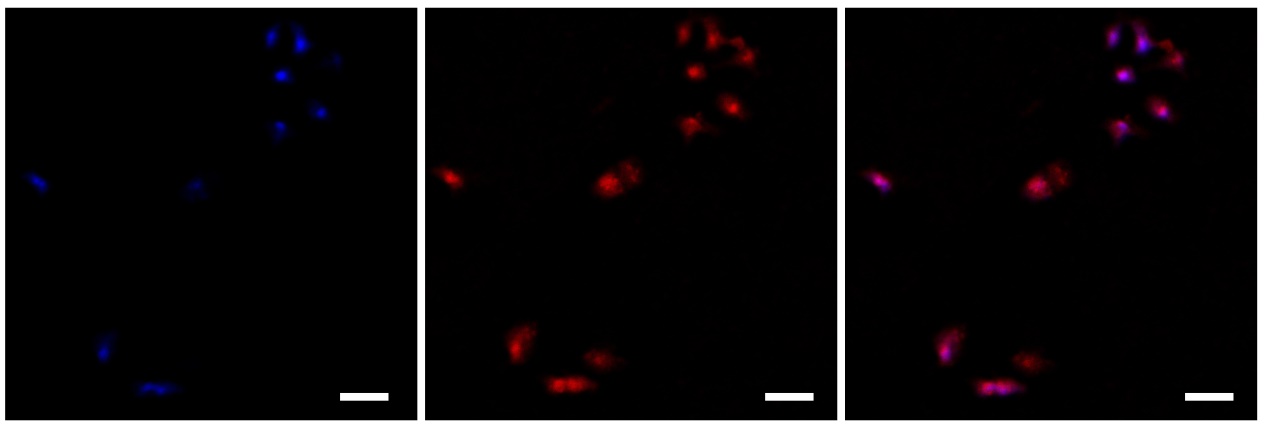


**Figure S6** The confocal laser scanning microscopy (CLSM) images of HeLa cells incubated with UCNP@mSiO_2_-Dopa.


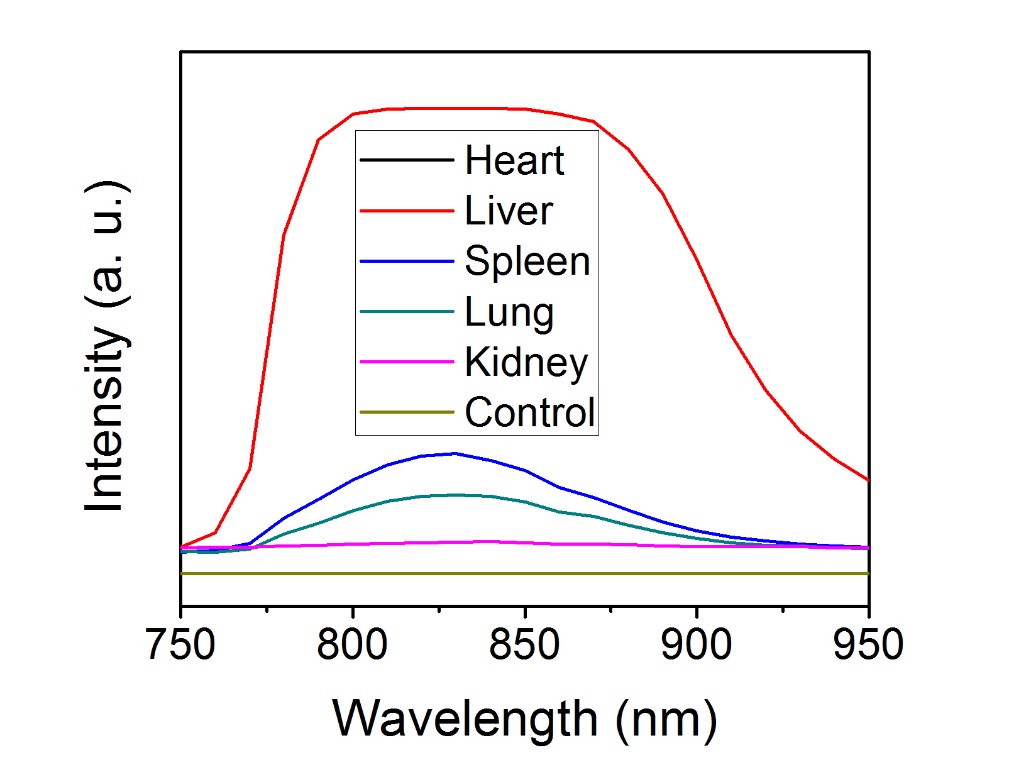


**Figure S7** Luminescence intensity of organs after intravenous injection of UCNP@mSiO_2_ incorporating ICG molecules for 2 hours.


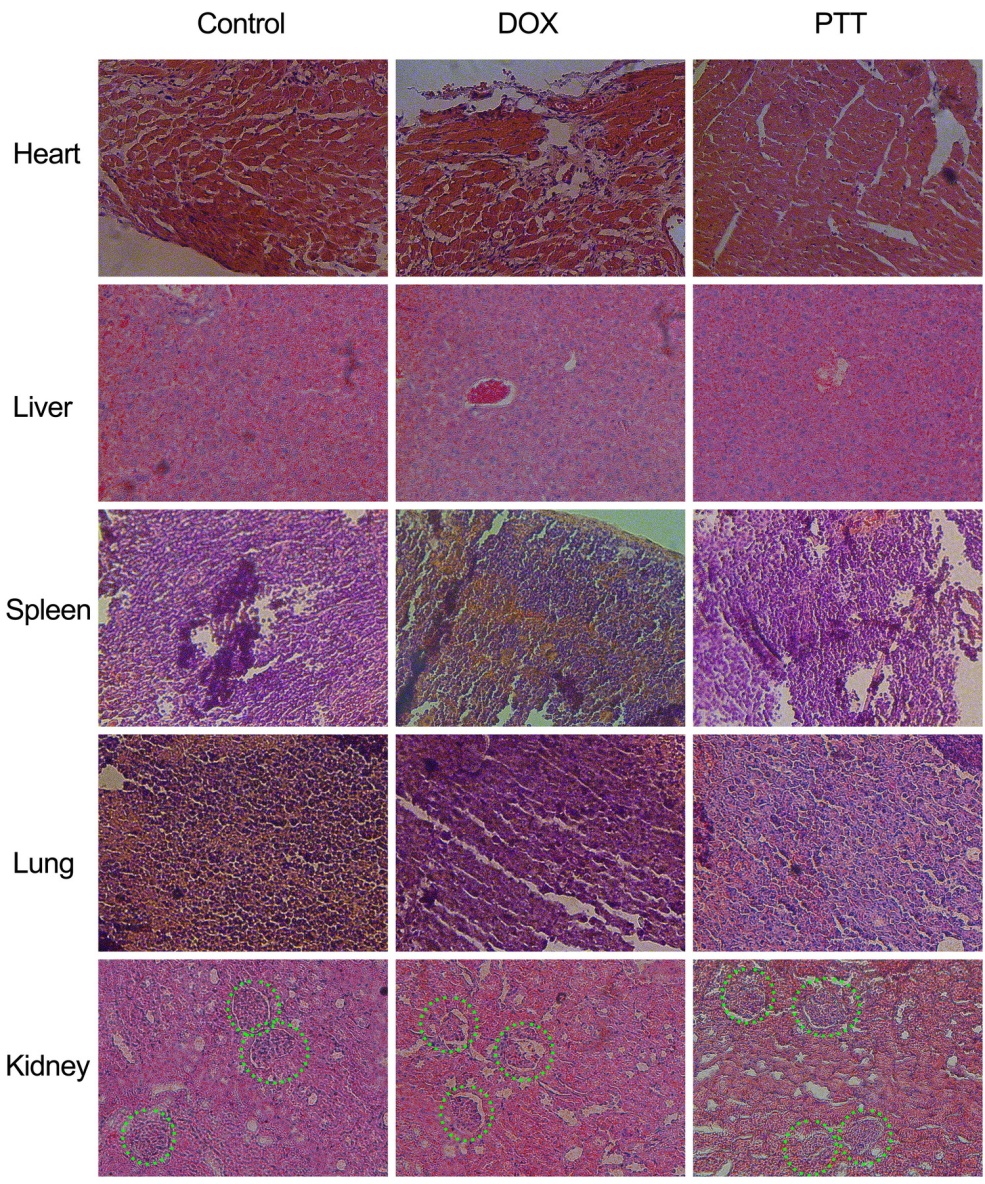


**Figure S8** H&E stained images of the tissues in the three groups.
